# CSF Neurofilament Light Chain but not FLT3 Ligand Discriminates Parkinsonian Disorders

**DOI:** 10.3389/fneur.2015.00091

**Published:** 2015-05-05

**Authors:** Megan K. Herbert, Marjolein B. Aerts, Marijke Beenes, Niklas Norgren, Rianne A. J. Esselink, Bastiaan R. Bloem, H. Bea Kuiperij, Marcel M. Verbeek

**Affiliations:** ^1^Department of Neurology and Parkinson Center, Donders Institute for Brain, Cognition and Behaviour, Radboud University Medical Centre, Nijmegen, Netherlands; ^2^Department of Laboratory Medicine, Radboud University Medical Centre, Nijmegen, Netherlands; ^3^Parkinson Center, Nijmegen, Netherlands; ^4^UmanDiagnostics, Umeå, Sweden

**Keywords:** Parkinson’s disease, multiple system atrophy, neurofilament light chain, FLT3 ligand, cerebrospinal fluid

## Abstract

The differentiation between multiple system atrophy (MSA) and Parkinson’s disease (PD) is difficult, particularly in early disease stages. Therefore, we aimed to evaluate the diagnostic value of neurofilament light chain (NFL), fms-like tyrosine kinase ligand (FLT3L), and total tau protein (t-tau) in cerebrospinal fluid (CSF) as biomarkers to discriminate MSA from PD. Using commercially available enzyme-linked immunosorbent assays, we measured CSF levels of NFL, FLT3L, and t-tau in a discovery cohort of 36 PD patients, 27 MSA patients, and 57 non-neurological controls and in a validation cohort of 32 PD patients, 25 MSA patients, 15 PSP patients, 5 CBS patients, and 56 non-neurological controls. Cut-offs obtained from individual assays and binary logistic regression models developed from combinations of biomarkers were assessed. CSF levels of NFL were substantially increased in MSA and discriminated between MSA and PD with a sensitivity of 74% and specificity of 92% (AUC = 0.85) in the discovery cohort and with 80% sensitivity and 97% specificity (AUC = 0.94) in the validation cohort. FLT3L levels in CSF were significantly lower in both PD and MSA compared to controls in the discovery cohort, but not in the validation cohort. t-tau levels were significantly higher in MSA than PD and controls. Addition of either FLT3L or t-tau to NFL did not improve discrimination of PD from MSA above NFL alone. Our findings show that increased levels of NFL in CSF offer clinically relevant, high accuracy discrimination between PD and MSA.

## Introduction

Parkinson’s disease (PD) is the most common movement disorder with typical age of onset around 60 years although some patients (~3–5%) develop PD before the age of 40 (1). PD is characterized by four cardinal motor features: involuntary tremor, postural instability, bradykinesia, and rigidity (2). Non-motor features such as cognitive disturbances, depression, mild autonomic dysfunction (including orthostatic hypotension), and disordered sleep commonly accompany these motor symptoms (3).

Multiple system atrophy (MSA) is a relatively rare and sporadic adult-onset disease characterized by a variable combination of parkinsonism, cerebellar ataxia, autonomic dysfunction (particularly orthostatic hypotension), and pyramidal signs (4). MSA is commonly misdiagnosed as PD, particularly in early disease stages, because of overlapping symptoms, occasionally good responsiveness to dopaminergic treatment in MSA, and similar age of onset, typically around 60 years (1, 4). However, MSA progresses more rapidly than PD and is associated with a much poorer quality of life (5). Moreover, the response to levodopa, although variable, is generally poor and may lead to worsening of orthostatic hypotension in some MSA patients (6). A reliable biomarker capable of clearly distinguishing between MSA and PD would have great clinical and diagnostic value.

Two recent studies have investigated the utility of fms-like tyrosine kinase ligand (FLT3L) as a potential cerebrospinal fluid (CSF) biomarker to differentiate MSA from PD but had conflicting results. While one paper reported that CSF levels of FLT3L could differentiate between MSA and PD with high accuracy (7) another showed no significant differences in FLT3L levels between MSA and PD (8). However, there is currently no literature available investigating the biological significance of FLT3L in PD. Therefore, additional studies are required to ascertain the utility of FLT3L as a biomarker and, if its utility in distinguishing PD from MSA can be confirmed, further studies investigating its biological significance would be highly warranted.

Neurofilament proteins are highly phosphorylated neuronal cytoskeleleton proteins composed of three subunits of which the smallest, the 68 kDa neurofilament light chain (NFL), forms the backbone and is essential for neurofilament assembly (9). Elevated levels of CSF NFL in atypical parkinsonisms compared with PD and controls have been observed and may reflect more extensive neuronal damage in AP than in PD. Similarly, tau has an important function in providing structural stability to axonal microtubules. Mutations in the MAPT gene have been associated with PD and thus mark tau as a potential biomarker for PD (10). As might be expected, CSF levels of NFL and total tau protein (t-tau) have been shown to discriminate PD from atypical parkinsonisms (11–14) but these studies require further validation. In the current study, we aimed to determine which CSF biomarker (NFL, t-tau, or FLT3L), or combination of biomarkers, could provide optimal discrimination of MSA from PD.

## Materials and Methods

### Patients

**Figure 1:**
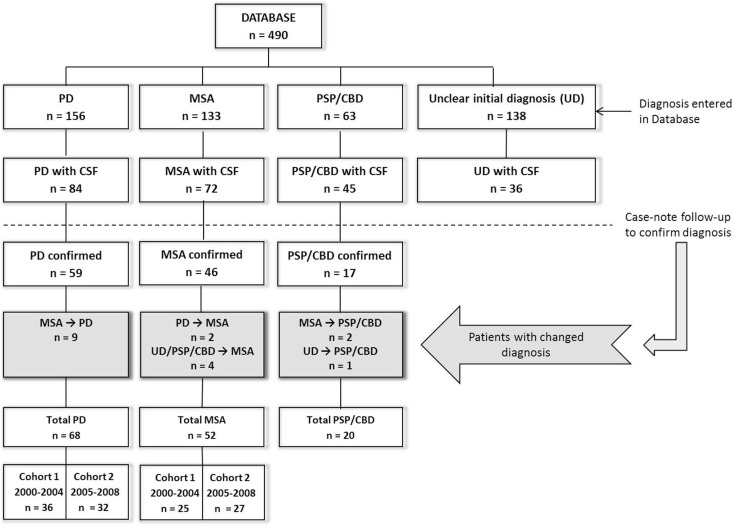
**Flowchart of patient inclusion in this study**. PD, Parkinson’s disease; MSA, multiple system atrophy; PSP, progressive supranuclear palsy; CBD, corticobasal degeneration; CSF, cerebrospinal fluid; *n*, number. CSF was obtained during the initial diagnostic assessment upon presentation.

The present study was performed at the Radboud University Medical Centre (Nijmegen, the Netherlands). We studied patients initially referred to our tertiary movement disorder center between December 2000 and November 2008 (Figure 1), with a hypokinetic rigid syndrome of uncertain diagnosis at presentation, and who received a subsequent diagnosis of PD or MSA. The initial clinical diagnosis (at presentation) was established by a neurologist specialized in movement disorders according to current diagnostic criteria for PD (15) and MSA (16). Patients underwent extensive neurological examination and a subset of the patients were included from a previous study in which they were studied prospectively for three years (57% of MSA and 86.8% of PD patients) (17). For these patients, diagnosis was established by two neurologists specialized in movement disorders and patients underwent extensive neurological examination and imaging studies at initial visit and again after 3 years (Supplementary Material). Ten MSA patients and five PD patients have been described earlier (14, 18) but the CSF parameters reported in the current study were not previously reported. The remaining eight MSA and four PD patients were incidental cases for whom case review follow-up was performed by a neurologist (author Marjolein B. Aerts).

Disease severity was established using the (modified) Hoehn and Yahr (H&Y) (19) stages and unified Parkinson’s disease rating scale (UPDRS) (20); ataxia severity was assessed using the International Cooperative Ataxia Rating Scale (ICARS) (21). Final diagnosis was confirmed by case review up to 9 years after initial visit. Controls consisted of patients referred to our Neurology Department during the period 2001–2009, who underwent lumbar puncture as part of the diagnostic process, and who had been confirmed as having no neurological disease.

For the discovery group, we analyzed CSF samples from PD and MSA patients obtained between 2001 and 2004, and controls consisted of patients with lumbar punctures obtained between 2001 and 2006. To validate our findings, we examined CSF in additional MSA and PD patients with lumbar punctures obtained between 2005 and 2008, and control CSFs obtained between 2007 and 2010. Additional patients diagnosed with progressive supranuclear palsy (PSP, *n* = 15) and corticobasal syndrome (CBS, *n* = 5) with previously unreported, retrospective CSF NFL levels were included to show differences in NFL levels between PD patients and other atypical parkinsonisms (Figure 1; Table S5 in Supplementary Material). Initial clinical diagnosis for these patients was established by a movement disorders specialist using current diagnostic criteria for PSP (22) and CBS (23). Lumbar puncture samples from all MSA and PD patients were analyzed for all CSF parameters to determine and validate the utility of these parameters in discriminating PD from MSA. Controls in the discovery group were tested for all CSF parameters for comparison with PD and MSA patients. NFL and FLT3L levels in a second group of controls in the validation cohort provided additional reference values for these parameters.

#### Ethical Statement

The following applies to 59/68 PD patients and 32/52 MSA patients: written informed consent was obtained from the participants prior to participation of the study. All clinical investigations have been conducted according to the principles expressed in the Declaration of Helsinki. In case patients were unable to consent (defined as MMSE score below 25), written informed consent was obtained from a next of kin of the patient. The local institutional review board (“Commissie Mensgebonden Onderzoek region Arnhem-Nijmegen”) approved of this study. For the remaining patients (i.e., 9/68 of PD patients, 20/52 MSA patients, and all controls), CSF samples were obtained as part of the clinical diagnostic work-up of a patient. Patients were informed that their data, including CSF, could be used for further scientific purposes and were given the option to object against this use, in which case their data were not used. This procedure has been approved as well.

### CSF Samples and analysis

Cerebrospinal fluid samples obtained by lumbar puncture were collected in polypropylene tubes, centrifuged (5 min, 860 × *g* at room temperature), and stored at −80°C. Patient information was decoded to maintain confidentiality. Undiluted CSF samples were measured in duplicate using commercially available enzyme-linked immunosorbent assays (ELISAs) for Human FLT3L (R&D Systems, Abingdon, UK), NFL (NF-light^®^ Neurofilament ELISA RUO; a gift from UmanDiagnostics, Sweden), and t-tau (INNOTEST^®^ hTau, Innogenetics N.V., Ghent, Belgium). ELISAs were performed according to manufacturer’s instructions except the capture antibody for the FLT3L ELISA was used at 1 μg/mL.

### Statistical analysis

Cerebrospinal fluid parameters with non-Gaussian distribution were log transformed and between-group differences were tested using one-way analysis of variance (ANOVA) followed by Tukey’s *post hoc* test. Mann–Whitney *U* tests were used to compare data with a non-Gaussian distribution (NFL levels in the discovery group). Spearman rank correlation was used to determine correlations. We performed analysis of covariance (ANCOVA) to control for possible confounding variables (e.g., age, gender, disease duration, and disease severity). Binary logistic regression was used to identify variables contributing to discrimination of MSA from PD and receiver–operator curves (ROCs) were used to determine the diagnostic accuracy of CSF parameters and models developed from the binary logistic regression. Statistical analyses were performed using GraphPad PRISM 5 software (San Diego, CA, USA) and SPSS software version 20.0 (Chicago, IL, USA). Comparison of the ROC curves was performed using MedCalc^®^ software version 12.5.0.0. Bootstrapping analyses using data from both cohorts were also performed for additional validation of the measures using Medcalc 12.7.0 9 Trial version.

## Results

### Patient characteristics

We analyzed 233 CSF samples: 52 MSA patients, 68 PD patients, and 113 non-neurological controls. Of these, 61% (32/52) of the MSA and 87% (59/68) of the PD patients had been studied prospectively for 3 years. The discovery group consisted of 36 PD, 27 MSA, and 57 controls. Patient characteristics and CSF parameters are reported in Table 1. CSF samples from controls were used to obtain reference values for NFL, FLT3L, and t-tau. In order to confirm the use of NFL, FLT3L, and t-tau in discriminating between PD and MSA, we included a validation group consisting of 32 PD and 25 MSA patients. Since CSF measures of NFL and FLT3L are rather novel, we included 56 additional controls to obtain additional reference values.

**Table 1:** **Patient demographic and baseline characteristics – discovery cohort**.

	PD (*n* = 36)	MSA (*n* = 27)	Controls (*n* = 57)
Age in years (SD)^b^	60.1 (10.4)	62.6 (9.0)	57.0 (11.5)
Number of males (%)	22 (61.1)	15 (55.6)	37 (64.9)
Years of follow-up (range)	5.5 (0–9.2)	2.9 (0–7.9)	N/A
NFL (ng/L)	1350 (915)	4548 (3206)	1503 (619)
FLT3L (ng/L)	38.4 (11.9)	39.3 (12.4)	47.8 (14.3)
t-tau (ng/L)	242 (190)	335 (164)	251 (110)
**Disease duration, months (range)^c^**	PD (*n* = 28)	MSA (*n* = 14)	***p*-Value^a^**
	43.8 (6–158)	43.7 (8–96)	*p* = 0.508
**Disease severity^c^**	PD (*n* = 32)	MSA (*n* = 14)	***p*-Value^a^**
H&Y	2.0 (0.60); *n* = 32	2.7 (1.2); *n* = 14	*p* = 0.014
UPDRS; mean (SD)	30.3 (11.5); *n* = 27	32.5 (16.7); *n* = 14	*p* = 0.627
ICARS; mean (SD)	2.9 (4.8); *n* = 20	12.1 (9.5); *n* = 9	*p* = 0.020
**Cognitive function**	PD (*n* = 28)	MSA (*n* = 14)	***p*-Value^a^**
MMSE; mean (SD)	28.5 (1.6)	27.5 (3.5)	*p* > 0.05

*SD, standard deviation; H&Y, Hoehn and Yahr score; ICARS, International Cooperative Ataxia Rating Scale; UPDRS, unified Parkinson’s disease rating scale; N/A, not applicable*.

*^a^Student’s *t*-test *p*-values for PD versus MSA*.

*^b^At time of lumbar puncture*.

*^c^At time of inclusion*.

### NFL, t-tau, and FLT3L levels in CSF of the discovery cohort

**Figure 2:**
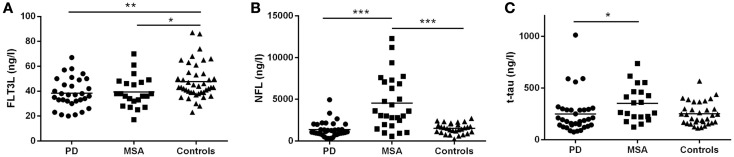
**Cerebrospinal fluid concentrations of FLT3L, NFL, and t-tau for the MSA, PD, and control groups: discovery cohort**. **(A)** FLT3L levels are significantly reduced in MSA and PD as compared to controls. NFL **(B)** and t-tau **(C)** concentrations are significantly increased in the MSA group compared with the PD group. MSA, multiple system atrophy; PD, Parkinson’s disease; NS, non-significant difference; mean values are indicated by horizontal lines.

In the discovery group, CSF FLT3L levels were significantly lower in PD (38.4 ± 11.9 ng/L; *p* < 0.01) and MSA (39.3 ± 12.4 ng/L; *p* < 0.05) compared with controls (47.8 ± 14.3 ng/L) but similar in MSA and PD (Figure 2A). We found significantly higher levels of CSF NFL in MSA (4548 ± 3206 ng/L) compared with both PD (1350 ± 915 ng/L, *p* < 0.001) and controls (1503 ± 619 ng/L, *p* < 0.001) but not between PD and controls (Figure 2B). CSF t-tau levels were significantly higher for MSA (335 ± 164 ng/L) than PD (242 ± 190 ng/L; *p* < 0.05; Figure 2C) and, compared with our reference values for t-tau in healthy controls, 46% of MSA patients and 11% of PD patients had elevated (≥350 ng/L) t-tau levels.

FLT3L correlated with both NFL and t-tau for PD and controls but not MSA. There was a moderate correlation between NFL and t-tau in PD (*r* = 0.39, *p* < 0.05) but not MSA (*r* = 0.34, *p* = 0.11) or controls (*r* = 0.43, *p* = 0.08). Details of the correlation data are provided in Table S1 in Supplementary Material. Four of the PD patients exhibited levels of t-tau that were markedly different from the remainder of the group (Figure 2C) but this was not correlated with MMSE since individual MMSE scores were 30 for 1 patient, 29 for 2 patients, and 26 for 1 patient. Despite long-term clinical follow-up (3–8.8 years), we can neither rule out, nor confirm, subclinical tauopathy in these patients.

Neurofilament light chain alone provided high discrimination (AUC 0.85) between MSA and PD with 74.1% sensitivity and 91.7% specificity. Logistic regression models of combination biomarkers were then analyzed (summarized in Table 2). The combination of t-tau and NFL developed in our previous study (12) (Model 1: *y* = NFL + 0.15*t-tau; AUC = 0.89) yielded similar sensitivity (75.0%) and specificity (91.2%; AUC = 0.90) for discriminating MSA from PD whereas the combination of FLT3L and NFL (Model 2: *y* = −1.646 + 0.001*NFL-0.0308*FLT3L) yielded a sensitivity of 81.8% and specificity of 94.8% (AUC = 0.89). The combination of NFL, FLT3L, and t-tau (Model 3: *y* = −3.054–0.001*NFL + 0.003*t-tau − 0.028*FLT3L) yielded a higher AUC (0.92) with increased sensitivity (94.7%) but reduced specificity (83.3%). Comparison of the ROC analyses showed that this improvement was not significantly better at discriminating between MSA from PD than NFL alone (*p* > 0.05).

**Table 2:** **Comparison of models for discriminating MSA from PD**.

CSF variables	Number of patients^a^	Cut-off ^b^ point	Sensitivity (%)	Specificity (%)	AUC	Youden index^c^	Likelihood ratio^d^
**DISCOVERY**							
NFL	PD = 36, MSA = 27	>2315	74.1	91.7	0.854	65.7	8.9
Model 1^e^	PD = 34, MSA = 20	>2388	75.0	91.2	0.879	70.6	8.5
Model 2^f^	PD = 31, MSA = 22	>−0. 925	81.8	94.8	0.887	68.9	6.3
Model 3^g^	PD = 29, MSA = 15	>−1.604	94.7	83.3	0.921	78.1	5.7
**VALIDATION**							
NFL	PD = 32, MSA = 25	>2315	80.0	96.9	0.938	76.9	25.6
Model 1^e^	PD = 32, MSA = 24	>2388	76.1	96.9	0.932	76.1	25.3
Model 2^f^	PD = 32, MSA = 23	>−0. 925	82.6	96.9	0.969	79.5	26.4
Model 3^g^	PD = 32, MSA = 22	>−1.604	81.8	96.9	0.948	75.6	26.2

*^a^Due to missing data points, not all CSF parameters were available in all patients*.

*^b^Cut-off refers to the selected value of the individual biomarker or the combination where the two groups can be separated at the indicated sensitivity and specificity*.

*^c^Youden index: sensitivity + specificity − 100*.

*^d^Likelihood ratio: sensitivity/(1 − specificity)*.

^e^Model 1: *y* = NFL + 0.15*t-tau.

^f^Model 2: *y * = −1.646 + 0.001*NFL-0.03*FLT3L.

^g^Model 3: *y* = −3.054–0.001*NFL + 0.003*t-tau − 0.028*FLT3L.

Gender was not correlated with CSF parameters for any group. Age was correlated with, or tended to be correlated with, all CSF parameters in PD and controls but not in MSA (Table S2 in Supplementary Material). Disease duration and severity (ICARS and H&Y) were not correlated with CSF parameters for either PD or MSA in the discovery cohort. UPDRS was not correlated with CSF parameters in the PD group but, intriguingly, showed a significant negative correlation with NFL (*r* = −0.57, *p* < 0.05) in MSA. Details of these correlations are provided in Table S3 in Supplementary Material. When we repeated our analyses controlling for age, gender, UPDRS, and disease duration using ANCOVA, significance levels were maintained for NFL but not FLT3L or t-tau, suggesting that NFL levels are robust but FLT3L and t-tau levels may be influenced by other factors that give rise to heterogeneous values.

### Validation of the diagnostic markers

In the validation cohort, we confirmed higher levels of CSF NFL in MSA (5938 ± 4267 ng/L) compared with PD (1103 ± 442 ng/L; *p* < 0.001) and controls (1290 ± 664 ng/L; *p* < 0.001; Table S4 in Supplementary Material). CSF NFL levels were also significantly higher in other atypical parkinsonsisms (AP; 15 PSP and 5 CBS) than in PD and controls (Table S5 in Supplementary Material). This significance was maintained after controlling for age, gender, and disease duration with AUC ≥ 0.9 (Figure S1 in Supplementary Material). FLT3L levels were non-significantly lower in both PD and MSA compared with the controls although a small significant difference between MSA and controls was found after controlling for age, gender, disease duration, and disease severity (*p* < 0.05). As with the discovery group, we also observed higher levels of t-tau in MSA than PD but this failed to reach significance (*p* = 0.06). We noted that t-tau levels in both PD and MSA in the validation groups were overall lower than in the discovery group and for MSA the difference in t-tau levels in discovery (335 ± 164 ng/L) versus validation (244 ± 93 ng/L) was significant (*p* < 0.05). The methodology used to measure t-tau (Innotest ELISAs) was the same for all patients but CSF samples collected prior to 2004 were analyzed retrospectively, which may have influenced our results.

Disease duration was significantly shorter in PD (25.1 months; range 6–84) than MSA (39.0 months; range 12–106) in the validation group, but controlling for this variable using ANCOVA did not alter the significance level for the CSF parameters.

The models developed using the CSF parameters from the discovery group were applied to the validation group and diagnostic values were calculated using cut-offs obtained from the discovery group. We could correctly identify the majority of MSA patients (sensitivity = 80% and specificity = 97%) using NFL alone (AUC = 0.94). Again, ROC curve comparison showed that none of the models significantly improved the discrimination of MSA from PD.

Bootstrapping analysis of the combined data to further validate our result, produced an ROC curve for NFL (PD versus MSA) that was highly comparable with ROC curves from the individual cohorts (AUC = 0.90; sensitivity = 77%; specificity = 96%, cut-off >2174 ng/L). Bootstrapping of the combined FLT3L data revealed significantly lower levels of FLT3L in both the PD and MSA groups compared with controls as was observed in the discovery group but not the validation group. We found no significant differences in CSF FLT3L levels between PD and MSA patients in the individual cohorts nor when using bootstrapping of the combined data.

## Discussion

In the current study, we showed that CSF levels of NFL can be used for clinically relevant discrimination of MSA from PD. These results confirm our previous findings using a different method of detection for NFL (14) and the findings of a more recent study using the same ELISA method (12). However, unlike these previous case-control studies, we recruited most of our patients from a prospective study with long clinical follow-up. Higher t-tau levels for MSA patients in this study confirm similar observation in other studies (12, 14, 18) but the contribution of t-tau to the overall discrimination of PD from MSA was not significant. We noted high t-tau values in around 11% of our PD patients and 46% of our MSA patients in the discovery group. However, very few patients in the validation group had high t-tau levels (3% of PD and 8% of MSA). Since the diagnostic value of our previously developed model combining NFL and t-tau (Model 1), did not differ between the discovery and validation groups, this variation probably did not adversely influence our results.

We found significantly decreased CSF FLT3L levels in PD and MSA compared to controls in our discovery cohort but not in the validation cohort. Bootstrapping of the combined data was consistent with the discovery group, revealing significantly lower levels of FLT3L in both PD and MSA compared with controls but we and others (10) found no significant differences in CSF FLT3L levels between PD and MSA. These results contradict an earlier study showing high accuracy discrimination between PD and MSA using FLT3L but we have used a different method of detection for FLT3L than the original paper (7) and our results agree with a more recent study using the same methodology as the original paper (9).

Unlike the first study (7), we did not attempt to exclude patients with possible familial PD, although genetic causes of PD were not identified in our cohorts. Variance could be partly attributable to inclusion of younger PD patients (<50 years in 15/52 PD patients) since we observed a strong correlation between age and FLT3L levels in both PD and controls. After subdivision of PD and MSA for age (i.e., >50 and <50 years), differences between PD and MSA were maintained and we found no differences between young versus old PD or MSA patients (data not shown).

Neurofilament proteins are essential for maintaining the neuronal cytoskeleton and increased levels of NFL in the CSF of MSA patients likely reflects extensive axonal degeneration. In keeping with earlier findings (12, 14), CSF NFL was increased in MSA and aided discrimination of MSA from PD and, in the current study, NFL alone provided the best tool for discriminating between MSA and PD. The addition of FLT3L and t-tau to NFL analysis improved this discrimination only slightly. However, we observed strong correlations between NFL and FLT3L in PD and controls in both the discovery and validation phases that warrant further investigation to determine the potential function of FLT3L in the central nervous system. The lack of correlation between NFL and FLT3L in the MSA patients suggests that increased levels of NFL were not dependent on changes in FLT3L or vice versa and does not support a role for FLT3L in the pathology of MSA.

FLT3L is a hematopoietic growth factor expressed in various tissues including the brain (24) and has an important role in hematopoietic stem cell survival and proliferation (25). Although FLT3L has a neurotrophic function contributing to increased survival of a subset of post-mitotic neurons (24), its role in neurodegenerative diseases is unknown. In amyotrophic lateral sclerosis (ALS), CSF levels of FLT3L are elevated compared with healthy controls (26). Nerve growth factor (NGF), which normally synergizes with FLT3L to exert its neurotrophic effect, also increases the expression of NFL. Since both NGF and NFL are increased in ALS (27, 28), NGF may contribute to elevated levels of FLT3L and NFL as observed in ALS (27, 29, 30). In PD, levels of NGF are reduced (29) and possibly contribute to observed reductions in CSF FLT3L and NFL levels in some patients. However, contrary to previous observations, CSF FLT3L levels alone do not serve as a biomarker for differentiation of MSA from PD.

A major strength of our study is that diagnosis was made prospectively for the majority of PD and MSA patients using detailed neurological examination in combination with imaging studies, and final diagnosis was confirmed after long follow-up by case review. Our findings emphasize a consistency with other studies (12–14, 31) showing that CSF NFL levels could be a useful adjunct to clinical diagnosis for distinguishing PD from MSA and other atypical parkinsonisms. Since both ours and previous studies have shown significantly increased CSF NFL levels in atypical parkinsonism disorders other than MSA, including PSP and CBS (12, 13, 31), CSF NFL levels do not represent a specific marker for MSA but rather, may be more generally useful for distinguishing PD from atypical parkinsonisms (12, 14). Our results will require confirmation in larger cohorts in future research, with (eventual) pathological confirmation of disease. Further, additional studies will also be required to determine whether NFL levels are influenced by other extraneous influences such as other non-neurological diseases (e.g., cancer) (32, 33) or familial versus sporadic forms of PD, and to determine whether increased NFL levels will be useful for differentiating PD from other APs at early stages of disease.

Following the successful identification of the CSF biomarkers t-tau, p-tau, and Aβ42 that support the clinical diagnosis of Alzheimer’s disease, there has been a growing interest in the discovery of similarly specific CSF biomarkers for PD and AP. Many studies have been reported on the quantification of α-synuclein in CSF. Although there is a general consistency that decreased concentrations of this protein are observed in PD and AP with α-synuclein pathology, large overlap between these patient groups and neurological controls has hindered the introduction of its quantification into clinical practice (34, 35). Similarly, a large degree of overlap is seen for levels of oligomeric α-synuclein and lysosomal enzyme levels in PD versus controls (36). Yet another approach, using proteomics discovery in CSF, did not yield a biomarker that could be applied in clinical practice since a panel of a minimum of five proteins was required to differentiate PD from controls at reasonable AUC (0.87) and the AUC of single markers did not exceed 0.79 (37). Therefore, a clinically useful CSF biomarker has not yet been identified for PD. In contrast, however, our study supports the concept that one (NFL) or two (NFL + t-tau) CSF biomarkers may reliably predict AP and PD in a population of patients with parkinsonism at a high AUC (>0.90). This combination of biomarkers also has a better clinical performance than the recently described CSF biomarker UCH-L1 (38), which differentiates PD from AP at an AUC of 0.69. In conclusion, currently the most progress has been made in identifying CSF biomarkers for AP, with NFL, either in combination with t-tau, being the most promising biomarker so far.

## Conflict of Interest Statement

Niklas Norgren is the CEO of UmanDiagnostics. UmanDiagnostics did not play any role in the study design and did not restrict or affect the data analysis in any way. The authors declare that the research was conducted in the absence of any commercial or financial relationships that could be construed as a potential conflict of interest.

## Supplementary Material

The Supplementary Material for this article can be found online at http://journal.frontiersin.org/article/10.3389/fneur.2015.00091
