# Supplementary material for: CSF Neurofilament Light Chain but not FLT3 Ligand Discriminates Parkinsonian Disorders
Source: Front Neurol. 2015 May 5;6:91. doi: 10.3389/fneur.2015.00091 (PMC4419719; doi:10.3389/fneur.2015.00091)

Supplementary Material

CSF neurofilament light chain but not FLT3 ligand discriminates Parkinsonian disorders

Megan K Herbert, PhD [a,b], Marjolein B Aerts, MD, PhD [a,c], Marijke Beenes [a,b], Niklas Norgren, PhD [d], Rianne A J Esselink, MD, PhD [a,c], Bastiaan R Bloem MD, PhD [a,c], H Bea Kuiperij, PhD [a,b], Marcel M Verbeek, PhD[a,b,c*]

[a] Department of Neurology and Parkinson Center, Donders Institute for Brain, Cognition and Behaviour, Radboud University Medical Centre, Nijmegen, the Netherlands
[b] Department of Laboratory Medicine, Radboud University Medical Centre, Nijmegen, the Netherlands
[c] Parkinson Center, Nijmegen, the Netherlands
[d] Uman Diagnostics, Umeå, Sweden

*** Correspondence:** Dr Marcel Verbeek, Department of Neurology, 830 TML, Neurochemistry Lab, Radboud University Medical Centre, P.O. Box 9101, 6500 HB Nijmegen, The Netherlands.
Marcel.Verbeek@radboudumc.nl

1. Supplementary Data

Methods

Prospective, longitudinal analysis of patients

During the period September 2003 until November 2006, consecutive new patients with a hypokinetic rigid syndrome who had been referred to the movement disorders clinic of the Department of Neurology at the Radboud University Medical Centre in Nijmegen, the Netherlands were recruited for a three year prospective study. Inclusion and exclusion criteria are provided in Table M1. Patients diagnosed with Parkinson's disease (PD) and multiple system atrophy (MSA) were identified for inclusion in the study.

Table M1. Inclusion and exclusion criteria

Inclusion criteria	Exclusion criteria
Hypokinetic rigid syndrome of neurodegenerative origin	Instable comorbidity
Aged > 18 years	Patients unfit to consent or participate

	Thrombopenia<br>A medical history of brain surgery

Informed consent was obtained and, within 6 weeks of the initial visit, all patients underwent a structured interview, detailed and standardized neurological examination, magnetic resonance imaging (MRI) scan, lumbar puncture, IBZM-SPECT and electromyography (EMG) of the anal sphincter.

Ethics

Medical ethics approval was obtained from the local Institutional Review Board (2002). All patients signed informed consent forms after detailed explanation of the procedures.

Interview and neurological examination

Interview and neurological examination were performed by two independent physicians, not directly involved in patient care. Using a structured interview the following items were assessed: medical history, current medications, presenting complaints and disease progression, most affected body site, balance and fear of falling, activities in daily living, and quality of sleep. In addition, the following clinimetric scales were scored: Unified Parkinson's Disease rating scale (UPDRS) III and IV and Hoehn and Yahr score,[1] International Cooperative Ataxia Rating Scale (ICARS),[2] Mini mental state examination (MMSE)[3] and frontal assessment battery (FAB) [4] for cognitive assessment, and Composite Autonomic Symptom Scale (COMPASS)[5] for autonomic dysfunction.

Follow up

Three years after the inclusion visit, patients were seen in the outpatient clinic for a repeated structured interview and neurological examination by an independent physician. These neurologists were blinded for the results of all ancillary investigations, except MRI which is

now nearly routinely used in clinical practice and to the clinical notes of the treating neurologist. A three year follow-up was chosen as previously published data show a very high concordance between neuropathological diagnosis and clinical diagnosis after at least 2 years follow up by a movement disorder specialist (PPV 99%) [6].

Clinical diagnosis

The clinical diagnosis was established in a systematic fashion by two movement disorder specialists, blinded for test results. Patient information was decoded to maintain patient confidentiality and sequentially presented to the panel in the following order: 1) clinical data and clinimetrics (UPDRS, MMSE, FAB, ICARS) upon inclusion, 2) disclosure of MRI results, 3) description of the reaction to dopaminergic medication and 4) disclosure of the clinical data and clinimetrics (UPDRS, MMSE, FAB, ICARS) after 3 years follow-up. Each time the panel -in consensus- established a diagnosis (either PD or AP (not otherwise specified) and the corresponding degree of uncertainty (on a 0-100% rating scale), followed by a more specific diagnosis (e.g. PD, MSA or PSP) always according to the international clinical criteria. **(**UK Parkinson's Disease Society Brain Bank clinical diagnostic criteria for PD,[7] NINDS-SPSP criteria for PSP, [8] Boeve criteria for CBS[9] McKeith Criteria for DLB [10] Gilman criteria for MSA [11] and Zijlmans criteria for VaP[12]. For the purposes of the current study, final diagnosis was confirmed by case review at extended (up to 9 years) follow-up.

Supplementary references

1. Hoehn MM, Yahr MD. Parkinsonism: onset, progression and mortality. Neurology. 1967 May;17(5):427-42.
2. Trouillas P, Takayanagi T, Hallett M, et al. International Cooperative Ataxia Rating Scale for pharmacological assessment of the cerebellar syndrome. The Ataxia Neuropharmacology Committee of the World Federation of Neurology. J Neurol Sci. 1997 Feb 12;145(2):205-11.
3. Folstein MF, Folstein SE, McHugh PR. "Mini-mental state". A practical method for grading the cognitive state of patients for the clinician. J Psychiatr Res. 1975 Nov;12(3):189-98.
4. Dubois B, Slachevsky A, Litvan I, Pillon B. The FAB: a Frontal Assessment Battery at bedside. Neurology. 2000 Dec 12;55(11):1621-6.

5. Suarez GA, Opfer-Gehrking TL, Offord KP, Atkinson EJ, O'Brien PC, Low PA. The Autonomic Symptom Profile: a new instrument to assess autonomic symptoms. Neurology. 1999 Feb;52(3):523-8.
6. Hughes AJ, Daniel SE, Ben-Shlomo Y, Lees AJ. The accuracy of diagnosis of parkinsonian syndromes in a specialist movement disorder service. Brain. 2002;125(4):861-70.
7. Hughes AJ, Daniel SE, Kilford L, Lees AJ. Accuracy of clinical diagnosis of idiopathic Parkinson's disease: a clinico-pathological study of 100 cases. Journal of neurology, neurosurgery, and psychiatry. 1992 Mar;55(3):181-4.
8. Litvan I, Agid Y, Calne D, et al. Clinical research criteria for the diagnosis of progressive supranuclear palsy (Steele-Richardson-Olszewski syndrome): report of the NINDS-SPSP international workshop. Neurology. 1996 Jul;47(1):1-9.
9. Boeve BF, Lang AE, Litvan I. Corticobasal degeneration and its relationship to progressive supranuclear palsy and frontotemporal dementia. Ann Neurol. 2003;54 Suppl 5:S15-9.
10. McKeith IG, Dickson DW, Lowe J, et al. Diagnosis and management of dementia with Lewy bodies: third report of the DLB Consortium. Neurology. 2005 Dec 27;65(12):1863-72.
11. Gilman S, Wenning GK, Low PA, et al. Second consensus statement on the diagnosis of multiple system atrophy. Neurology. 2008 August 26, 2008;71(9):670-6.
12. Zijlmans JC, Daniel SE, Hughes AJ, Revesz T, Lees AJ. Clinicopathological investigation of vascular parkinsonism, including clinical criteria for diagnosis. Movement disorders : official journal of the Movement Disorder Society. 2004 Jun;19(6):630-40.

2. Supplementary Figures and Tables

Supplementary Tables

Table 1. CSF parameter correlations

Diagnosis			NFL	t-tau
PD	*Discovery*	FLT3L	r=0.479, p<0.01	r=0.671, p<0.001
		NFL		r=0.392, p<0.05
	Validation	FLT3L	r=0.520, p<0.01	r=0.400, p<0.05
		NFL		r=0.524, p<0.01
MSA	*Discovery*	FLT3L	r=0.024, *ns*	r=0.320, *ns*
		NFL		r=0.39, *ns*
	Validation	FLT3L	r=0.334, *ns*	r=0.337, *ns*
		NFL		r=0.299, *ns*
Control	*Discovery*	FLT3L	r=0.870, p<0.001	r=0.502, p<0.01
		NFL		r=0.434, p=0.08 [a]
	Validation	FLT3L	r=0.491, p=0.001	r=-0.775, p<0.05
		NFL		r=-0.500, *ns*

ns: not significant (p<0.1); [a] values showing a trend where p=0.05 to 0.1.

Table 2. **Age correlations with CSF parameters**

Diagnosis		FLT3L	NFL	t-tau
PD	*Discovery*	r=0.406, p <0.05	r=0.706, p<0.001	r=0.359, p<0.05
	Validation	r=0.469, p<0.01	r=0.788, p<0.001	r=0.544, p<0.01
MSA	*Discovery*	r=0.219, *ns*	r=-0.079, *ns*	r=0.404, *p*=0.051 [a]
	Validation	r=0.248, *ns*	r=-0.208, *ns*	r=0.122, *ns*
Controls	*Discovery*	r=0.289, p=0.064 [a]	r=0.599, p<0.01	r=0.612, p<0.001
	Validation	r=0.149, *ns*	r=0.540, p<0.001	r=-0.071, *ns*

ns: not significant (p<0.1); [a] values showing a trend where p=0.05 to 0.1.

Table 3. **Correlations between CSF parameters and disease duration and severity**

Diagnosis			FLT3L	NFL	t-tau
PD	*Discovery*	Disease duration	r=0.235, *ns*	r=0.010, *ns*	r=-0.027, *ns*
		UPDRS	r=0.191, *ns*	r=-0.143, *ns*	r=-0.160, *ns*
		ICARS	r=-0.452, *ns*	r=-0.211, *ns*	r=-0.262, *ns*
		H&Y	r=-0.036, *ns*	r=0.089, *ns*	r=-0.160, *ns*
	Validation	Disease duration	r=0.215, *ns*	r=0.139, *ns*	r=0.136, *ns*
		UPDRS	r=0.118, *ns*	r=0.348, p=0.06 [a]	r=0.094, *ns*
		ICARS	r=0.361, p=0.05 [a]	r=0.146, *ns*	r=0.047, *ns*
		H&Y	r=0.116, *ns*	r=0.296, *ns*	r=0.214, *ns*
MSA	*Discovery*	Disease duration	r=0.277, *ns*	r=0.268, *ns*	r=0.454, p=0.07 [a]
		UPDRS	r=-0.041, *ns*	r=-0.570, p<0.05	r=-0.524, p=0.06 [a]
		ICARS	r=-0.120, *ns*	r=0.151, *ns*	r=0.159, *ns*
		H&Y	r=0.136, *ns*	r=-0.161, *ns*	r=-0.389, *ns*
	Validation	Disease duration	r=-0.267, *ns*	r=-0.196, *ns*	r=-0.478, p<0.05
		UPDRS	r=0.116, *ns*	r=0.122, *ns*	r=-0.010, *ns*
		ICARS	r=0.266, *ns*	r=0.455, *ns*	r=0.273, *ns*
		H&Y	r=0.079, *ns*	r=0.018, *ns*	r=-0.055, *ns*

ns: not significant (p<0.1); [a] values showing a trend where p=0.05 to 0.1.

Table 4. Patient demographic and baseline characteristics for validation cohort

	PD (n= 32)	*MSA (n=25)*	*Controls (n=56)*
Age in years (SD)[x]	56.5 (11.7)	62,5 (9.5)	55.9 (11.1)
Number of males (%)	23 (71.9%)	17 (68.0%)	32 (57.1%)
Years of follow-up (range)	4.3 (0-7.1)	3.4 (0-7.4)	N/A
NFL (ng/L)	1103 (442)	5938 (4267)	1290 (664)
FLT3L (ng/L)	37.4 (9.4)	35.8 (9.5)	41.3 (10.7)
t-tau (ng/L)	198 (74)	237 (93)	193 (53)
**Disease duration,**			***p*-value[a]**
**months (range)[†]**	25.1 (6 - 84)	38.0 (12 – 106)	$p = 0.06$
**Disease severity** [†]			
H&Y	1.7 (0.4); n=30	2.6 (0.9); n=18	$p < 0.01$
UPDRS; mean(SD)	20.4 (8.9); n=30	32.4 (13.7); n=17	$p < 0.01$
ICARS; mean(SD)	2.6 (4.8); n=30	10.9 (2.6); n=18	$p < 0.001$

SD: standard deviation; H&Y: Hoehn and Yahr score; ICARS: International Cooperative Ataxia Rating Scale; UPDRS: Unified Parkinson's Disease Rating Scale; N/A: not applicable

[a] Student's t-test *p*-values for PD versus MSA; [x] At time of lumbar puncture; [†]At time of inclusion.

Table 5. Demographic characteristics of PSP and CBS patients

	PSP (n= 15)	*CBS (n=5)*
Age in years (SD)[x]	67.2 (7.3)[*]	65.5 (8.5)
Number of males (%)	9 (60%)	3 (60%)
Years of follow-up (range)	2.4 (0-6.3)	2.1 (0.3-2.5)
NFL (ng/L)	4524 (1666)[***]	3990 (3495)[#]
**Disease duration, months (range)**[†]	27.4 (14-38), *n=7*	NA
**Disease severity** [†]		
H&Y	2.9 (0.53); *n=7*	NA
UPDRS; mean(SD)	31.1 (10.1); *n=7*	NA
ICARS; mean(SD)	9.2 (5.2); *n=7*	NA

[*]p<0.05, [***]p<0.001, [#]p=0.087 compared with PD; NA: not available

Figure 1. Receiver operator curve analysis for PD vs. (combined) MSA, PSP and CBS groups:

ROC A: comparison between PD and MSA

ROC B: comparison between PD and combined PSP and CBS groups

ROC C: PD versus all atypical parkinsonisms (MSA, PSP and CBS; ROC C) for the combined data.

ROC A (AUC = 0.0.90) **ROC B (AUC = 0.91)** **ROC C (AUC = 0.95)**

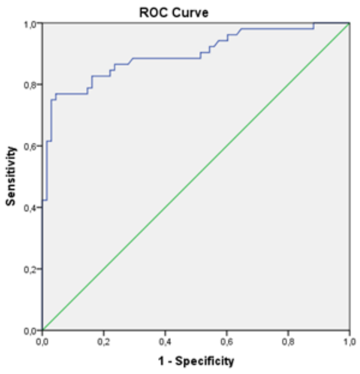

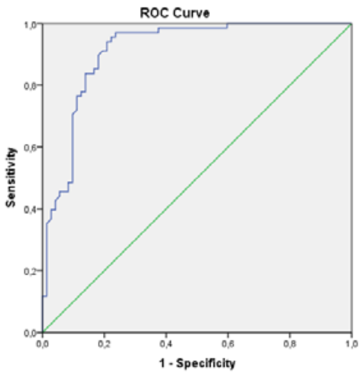

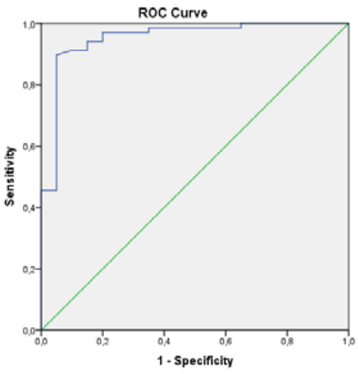

Supplement: Supplementary file 1 [file Data_Sheet_1.PDF]
